# A Novel Transformer-Based Multivariate Spatio-Temporal Feature Fusion Method for UAV Actuator Anomaly Detection

**DOI:** 10.3390/s26175467

**Published:** 2026-08-29

**Authors:** Chenyu Liu, Hao Yue

**Affiliations:** 1 North Alabama International College of Engineering and Technology, Guizhou University, Guiyang 550025, China; 2 State Key Laboratory of Public Big Data, Guizhou University, Guiyang 550025, China

**Keywords:** unmanned aerial vehicle (UAVs), actuator, multivariate signal, feature fusion, anomaly detection

## Abstract

The actuator plays a crucial role in controlling the flight attitude of unmanned aerial vehicles (UAVs), making timely anomaly detection essential for operational reliability and flight safety. However, existing methods often have difficulty jointly modeling the complex temporal dynamics and inter-variable spatial dependencies of multivariate actuator signals, while their computational complexity can limit real-time deployment. Moreover, most existing approaches rely on univariate or weakly coupled representations, making them less effective in detecting simultaneous faults across multiple actuators. To address these challenges, this paper proposes a Multivariate Spatio-Temporal Feature Fusion Transformer (STF_Tran) framework for anomaly detection in fixed-wing UAV actuators. Unlike conventional transformer-based multivariate anomaly detection methods that employ a shared attention mechanism to model heterogeneous dependencies, STF_Tran adopts a dual-branch architecture that separately encodes temporal dynamics and spatial correlations from multivariate actuator signals. A self-learning mechanism enables each branch to learn discriminative representations directly from normal operating data without requiring explicit fault labels, while a feature fusion module integrates the complementary spatio-temporal representations for anomaly reconstruction and scoring. Faults are identified by comparing the resulting anomaly scores with predefined thresholds, enabling the detection of diverse and simultaneous actuator anomalies. Experimental results demonstrate the effectiveness of STF_Tran, achieving F1 scores of 0.9944 and 0.9949 for deviation and stuck anomalies, respectively, and consistently outperforming several state-of-the-art methods.

## 1. Introduction

In recent years, the rapid development of the unmanned aerial vehicles (UAVs) has led to their widespread adoption across diverse sectors, including surveillance [1], agriculture [2], and military applications [3]. Among various types, fixed-wing UAVs are particularly valued for their high payload capacity and long-range flight capabilities, making them well-suited for a broad spectrum of missions [4,5]. The core of UAV flight control lies the actuator, a key component responsible for managing attitude, executing control commands, and enabling complex maneuvers. The reliable functioning of actuators is therefore essential to mission success. However, actuator failures can not only compromise task execution but also pose serious threats to public safety under complex conditions [6,7]. As such, advanced condition monitoring and timely detection of potential actuator anomalies are critical for enhancing the reliability and safety of UAV operations.

The primary goal of UAV anomaly detection is to identify data patterns that deviate from established norms, indicating potential system faults or irregularities [8]. Existing methods can generally be categorized into three main approaches [9]: (1) Knowledge-based methods, which rely on expert knowledge and predefined rules but show certain limitations in adapting to novel or evolving scenarios. Previous studies have incorporated adaptive neural networks and particle filtering (PF) for estimating state residuals, as well as fuzzy inference systems (FIS) for decision-making in anomaly detection tasks [10,11]. (2) Model-based methods, which perform anomaly detection by constructing mathematical models of UAV subsystems and have strong real-time performance. However, their effectiveness depends on the accuracy of physical models and cannot be used for complex situations. For instance, Estrada et al. [12] developed a linearly varying parameter (LPV) system for anomaly detection. Similarly, Suarez et al. [13] designed a fault detection, identification, and recovery framework using data from position, attitude, and vision sensors. Yang et al. [14] applied Extended Kalman Filtering (EKF) to detect anomalies in nonlinear UAV dynamic models.

In contrast to knowledge-based and model-based approaches, data-driven methods focus on leveraging historical data to train machine learning and deep learning models to learn feature patterns from UAV sensor data. These approaches have become mainstream in UAV anomaly detection due to their ability to achieve high accuracy without the need to construct complex physical models [15]. For example, Bronz et al. [16] applied Support Vector Machines (SVMs) to map data into a higher-dimensional space, thereby enhancing anomaly detection accuracy. Alos [17] and Liang [18] employed Principal Component Analysis (PCA) and Kernel Principal Component Analysis (KPCA), respectively, to reduce data dimensionality and improve detection performance. Jeon et al. [19] proposed a hybrid model combining Long Short-Term Memory (LSTM) networks with autoencoders (AE) for effective structural anomaly detection in UAVs. Wang et al. [20] further advanced the field by integrating transformers, graph attention mechanisms, and multi-channel data fusion for multivariate anomaly detection. Moreover, Deng [21] proposed a method based on sensor data fusion and a hybrid multimodal neural network, addressing the inherent interdependencies among multiple UAV sensors. Wang et al. [22] developed an anomaly detection method using LSTM-based prediction, identifying anomalies by comparing predicted values with predefined uncertainty intervals. Zhong et al. [23] applied artificial neural networks (ANN) to model parameter correlations and utilized LSTM-based regression, employing threshold and residual analysis for anomaly detection.

Despite the performance of data-driven approaches in UAV anomaly detection, several challenges persist. One of the primary difficulties lies in the high cost and complexity of collecting UAV anomaly data, coupled with the challenge of accurately labeling such data. These factors significantly constrain the effectiveness of supervised learning methods [16,21]. As a result, there is growing interest in unsupervised anomaly detection techniques, which aim to identify deviations from normal behavior without relying on labeled data, an approach that aligns well with emerging research trends [24]. Moreover, UAV sensor data inherently exhibits rich spatio-temporal characteristics, but many existing methods tend to overlook either the temporal dimension [16,17,20,21] or the spatial structure [22,25,26]. While LSTM networks are widely used to extract temporal features [22,23,27], their computational overhead can be substantial and may fall short in meeting the real-time requirements of UAV anomaly detection tasks [28]. In addition, current methods focus on detecting anomalies in individual variables, despite the fact that UAV systems are composed of numerous interrelated parameters. This gap limits the ability to capture complex system-wide anomalies, making single-variable detection approaches inadequate for ensuring comprehensive and reliable anomaly identification. More importantly, existing multivariate anomaly detection methods often rely on shared or correlation-based representations, without explicitly disentangling temporal dynamics from cross-variable spatial dependencies. Although transformers offer powerful dependency modeling, their application to UAV anomaly detection often overlooks this spatio-temporal decoupling and real-time efficiency. Therefore, an unsupervised framework is needed to independently learn and efficiently fuse temporal and spatial representations for detecting anomalies across correlated actuator variables.

To address the aforementioned challenges, we propose a Multivariate Spatio-Temporal Feature Fusion Transformer (STF_Tran) method for fixed-wing UAV actuator anomaly detection. Unlike existing methods that primarily depend on correlation analysis to capture parameter-level spatio-temporal relationships [22,23], our approach utilizes a transformer architecture with two dedicated sub-encoders that independently extract temporal and spatial features. A linear layer is incorporated to efficiently capture temporal patterns within a specified time window, enhancing both feature quality and inference speed. These temporal features are then fused with spatial features via the Hadamard product, resulting in enriched spatio-temporal representations. Finally, the fused features are decoded to generate multivariate reconstruction outputs, and anomaly detection is achieved by comparing the resulting anomaly scores with a predefined threshold to identify deviations in UAV actuator behavior. The actuator-specific novelty of STF_Tran lies in its ability to jointly model multiple coupled fixed-wing UAV actuator signals rather than treating each actuator variable independently. In this study, the Elevator, Aileron, left elevon, and right elevon are considered as a correlated actuator subsystem, and STF_Tran captures both their temporal dynamics and cross-variable spatial dependencies for anomaly detection. The main contributions of this paper can be summarized as follows:

(1) A Multivariate Spatio-Temporal Feature Fusion Transformer framework is proposed for multivariate anomaly detection in fixed-wing UAV actuators.

(2) A dual-subnetwork architecture is designed to separately extract and fuse spatial and temporal features, enabling more effective feature representation.

(3) The method is validated on real UAV actuator data with injected anomalies through comprehensive experiments, proving its robustness and effectiveness.

The rest of the paper is structured as follows: Section 2 describes the details of the proposed method, Section 3 introduces the dataset and experimental setups, Section 4 analyzes the experimental results and Section 5 discusses the key parameters. Finally, Section 6 summarizes this work and discusses the future directions.

## 2. Methodology

**Figure 1:**
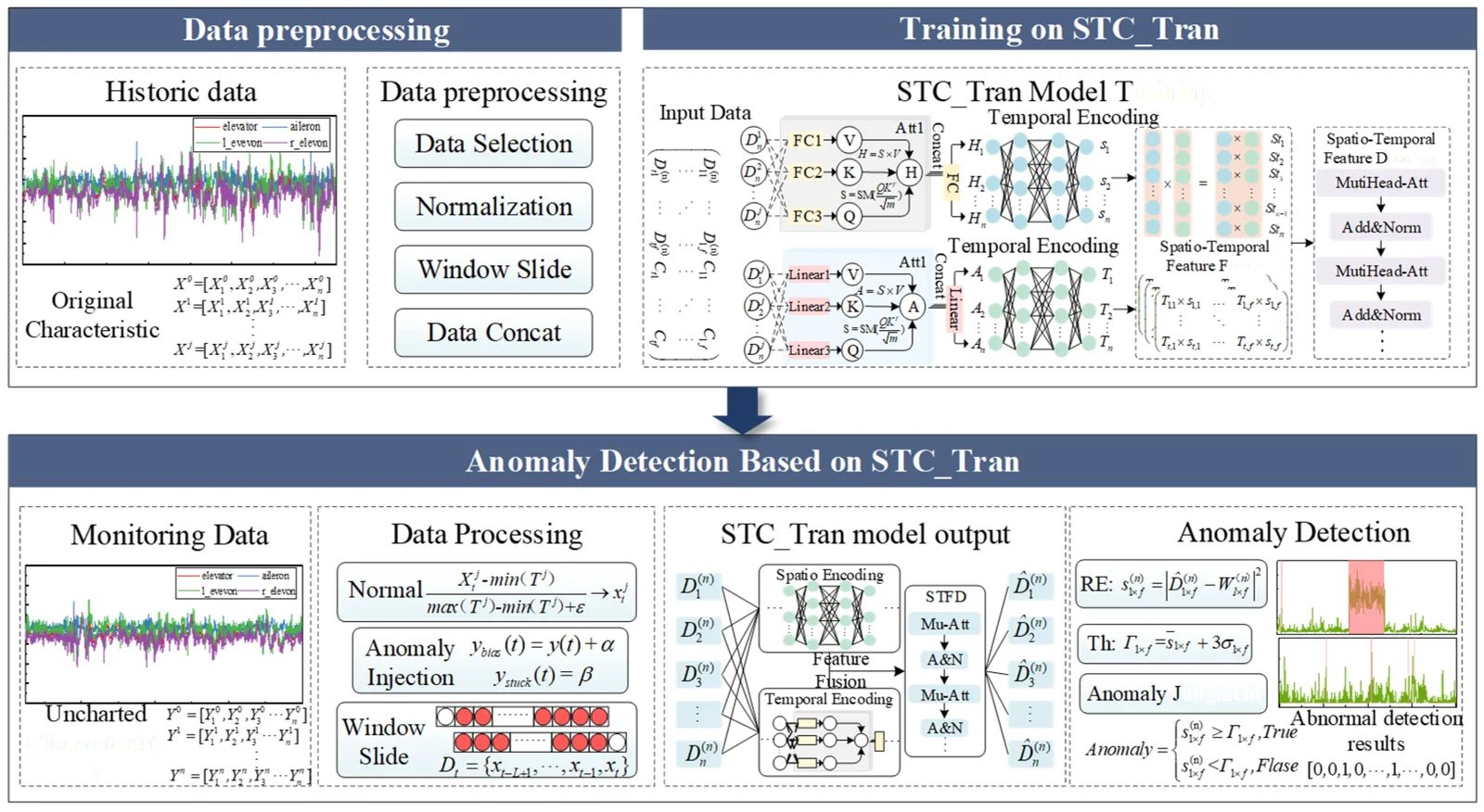
The proposed STF_Tran framework.

In this section, we present the proposed STF_Tran model for detecting anomalies in UAV actuators. Figure 1 illustrates the overall framework, which comprises three primary phases: (1) Data preprocessing: this process begins with data normalization to reduce the impact of parameter range discrepancies on feature learning, and a sliding window technique is then applied to capture contextual information. (2) Training stage: the preprocessed data is embedded into the input and subsequently fed into the STF_Tran model for training, where two subnetworks extract and fuse spatial and temporal features, respectively. (3) Anomaly detection stage: the preprocessed data is then input into the trained STF_Tran model to compute anomaly scores, and anomalies are identified by comparing these scores against a predefined threshold. This three-stage framework enables robust and effective detection of anomalies in multidimensional UAV actuator data. The proposed STF_Tran is an unsupervised, reconstruction-based deep learning algorithm for multivariate UAV actuator anomaly detection. It learns normal spatio-temporal patterns from actuator time-series data without requiring labeled anomaly samples, reconstructs the actuator variables, and identifies abnormal states according to reconstruction-based anomaly scores.

### 2.1. Data Preprocessing

#### 2.1.1. Normalization

Since the value ranges of UAV actuator parameters vary significantly, the first step involves normalizing the data and converting it into time-series windows, which ensures that the model can accurately learn features during both training and testing phases. Let the input multidimensional time-series data be denoted as $T={\left\{{\left\{X_{tj}\right\}}_{i=1}^{N}\right\}}_{j=1}^{M}$, where t denotes the tth moment and i∈N, j denotes the jth parameter, and $X_{t}^{j}$ represents the value of the jth parameter at the tth moment. The normalization formula is as follows:

(1) $$\frac{X_{t}^{j}-\min\left(T^{j}\right)}{\max\left(T^{j}\right)-\min\left(T^{j}\right)+\varepsilon }\to x_{t}^{j}$$

where $\min\left(T^{j}\right)$ denotes the minimum value of the jth parameter of the multidimensional sequence T, $\max\left(T^{j}\right)$ is the maximum value of the jth parameter of the multidimensional sequence T, $x_{t}^{j}$ denotes normalized data, and ε is the smallest constant that avoids a denominator of 0. Equation (1) allows T to be normalized to data in the range [0, 1].

#### 2.1.2. Sliding Window

To capture the contextual representation of the time point $x_{t}^{j}$, a sliding window method with a length of L is applied to the data, generating time-series segments for subsequent processing:

(2) $$D_{t}=\left\{x_{t-L+1},\ldots ,x_{t-1},x_{t}\right\}$$

where $x_{t}$ is a multivariate vector, not a single scalar, for time points where t<L, a replication strategy, is employed to preserve the fixed window length L each time step. Through this process, the original multidimensional time-series T is transformed into sliding window data. Rather than directly using T as the model input, we utilize the derived windowed dataset D, which is associated with T as the training data for the model.

### 2.2. Model Structure

**Figure 2:**
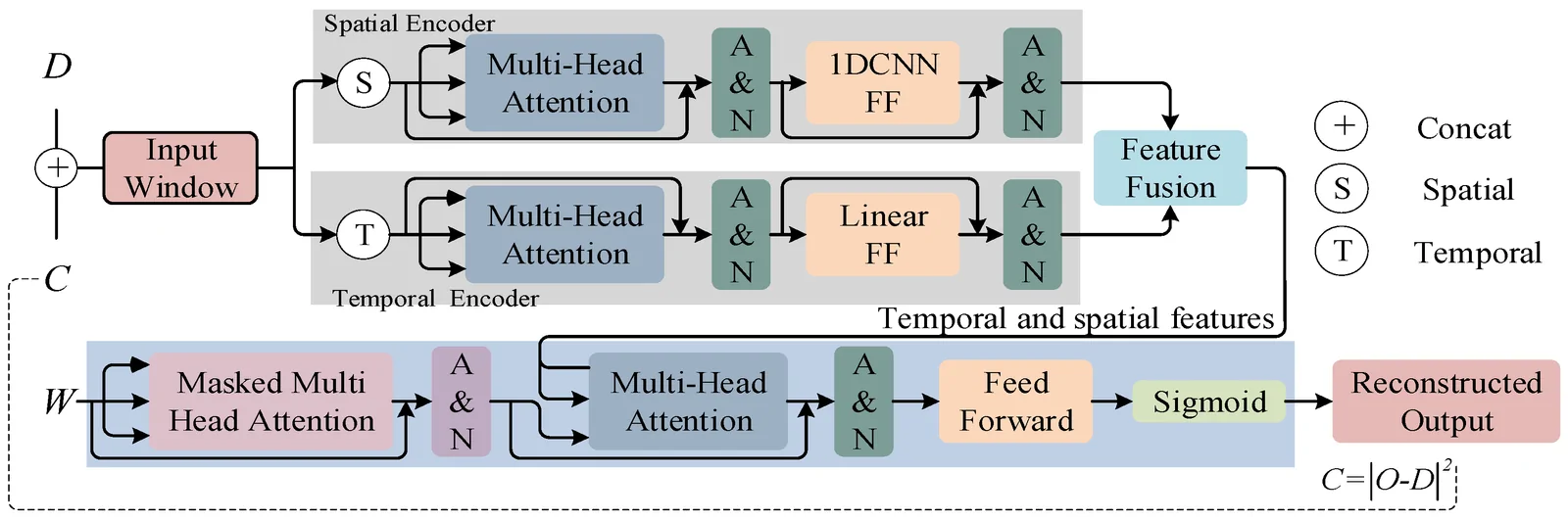
The architecture of the proposed STF_Tran.

Recently, the transformer model has gained significant attention as one of the most popular deep learning architectures, showing distinct advantages across various domains [29], particularly effective in capturing complex interdependencies within time-series data. Through the multi-attention mechanism, they emphasize key and interconnected features in the input, thereby enhancing the model’s ability to process global information. The transformer architecture can be divided into four primary modules: the input module, output module, encoding module, and decoding module. The traditional transformer mainly consists of a feedforward neural network and a multi-attention mechanism, with the latter serving as its core component [29]. To meet the intricate demands of multivariate anomaly detection for UAV actuators, a novel adaptation of the transform theory has been developed, complemented by targeted enhancements, as illustrated in Figure 2.

Compared with the traditional data input of transformers, this paper adopts the introduction of a new point-in-time variable *C* associated with the input data and concatenates C with the input data to enrich the feature information of the input data. Assume that the preprocessed data is $D=\left\{D_{t}^{\left(1\right)},D_{t}^{\left(2\right)},\ldots ,D_{t}^{\left(m\right)}\right\}$, $D_{t\times f}^{\left(n\right)}$ is the window data of the nth, t denotes the window length, and f is the feature length. First connect $D_{t\times f}^{\left(n\right)}$, with c in series:

(3) $$D_{t\times 2f}^{\left(n\right)}=D_{t\times f}^{\left(n\right)}C_{t\times f}=\left(\begin{array}{cccccc} D_{11}^{\left(n\right)} & \ldots & D_{1f}^{\left(n\right)} & C_{11} & \ldots & C_{1f}\\ \vdots & \ddots & \vdots & \vdots & \ddots & \vdots \\ D_{t1}^{\left(n\right)} & \ldots & D_{tf}^{\left(n\right)} & C_{t1} & \ldots & C_{tf} \end{array}\right)$$

where $D_{t\times 2f}^{\left(n\right)}$ represents the data after concatenation.

#### 2.2.1. Spatial Feature Encoding

Input data first undergoes spatial encoding to learn critical spatial features, utilizing a multi-head attention mechanism integrated with a ffeedforwardneural network. Unlike the conventional transformer model that employs a fully connected layer for the feed-forward network, this study adopts a 1D convolutional neural network (1DCNN). The choice of 1DCNN stems from its superior feature extraction capability and its ability to process time-series data directly without requiring dimensional conversion. Spatial encoding emphasizes capturing parameter relationships within the input window data, effectively extracting interdependencies among different parameters. The spatial feature encoding is computed as follows:

(4) $$Q_{t\times q}^{i}=D_{t\times 2f}^{\left(n\right)}\times W_{f\times q}^{Q}+b_{t\times q}^{Q}$$

(5) $$K_{t\times q}^{i}=D_{t\times 2f}^{\left(n\right)}\times W_{f\times q}^{K}+b_{t\times q}^{K}$$

(6) $$V_{t\times q}^{i}=D_{t\times 2f}^{\left(n\right)}\times W_{f\times q}^{V}+b_{t\times q}^{V}$$

where t denotes the length of the input window data, q denotes the mapped feature dimension, f is the feature dimension of the input, $Q_{t\times q}^{i},\;K_{t\times q}^{i},\;V_{t\times q}^{i}$ denote the Query, Key, and Value computed from the same input, and $W_{f\times q}^{Q},\;W_{f\times q}^{K},\;W_{f\times q}^{V}$ represent the weight matrices of Query, Key, and Value, respectively. $b_{f\times q}^{Q},\;b_{f\times q}^{K},\;b_{f\times q}^{V}$ denote the bias matrices of Query, Key, and Value. By utilizing the obtained values of $Q_{t\times q}^{i},\;K_{t\times q}^{i},\;V_{t\times q}^{i}$, the attention mechanism for the ith element can be computed:

(7) $$H_{t\times t}^{i}=\mathrm{Attention}(Q_{t\times q}^{i},K_{t\times q}^{i},V_{t\times q}^{i})=\mathrm{softmax}\left(\frac{Q_{t\times q}^{i}{K_{t\times q}^{i}}^{⊤}}{\sqrt{q}}\right)V_{t\times q}^{i}$$

where $Q_{t\times q}^{i}{K_{t\times q}^{i}}^{⊤}$ denotes dot product to compute similarity, $\sqrt{q}$ scales the similarity matrix to speed up convergence, Softmax converts the similarity matrix between [0, 1], and $H_{t\times q}^{i}$ is used to get the attention matrix. Since the multi-head attention mechanism contains multiple attention matrices, multiple attention matrices are obtained by concatenating them:

(8) $$\mathrm{MultiHeadAtten}=\mathrm{Concat}(H_{t\times q}^{1},H_{t\times q}^{2},\ldots ,H_{t\times q}^{i})=H_{t\times q}$$

where $H_{t\times q}$ is the combined multi-head attention score. It is also necessary to use the full connection layer to reconstruct the multi-head attention scores into the required dimensions:

(9) $$H_{t\times 2f}=F(H_{t\times iq})=H_{t\times iq}*W_{iq\times 2f}$$

where F(⋅) is $H_{t\times iq}\to H_{t\times 2f}$; $W_{iq\times 2f}$ is the weight matrix. $H_{t\times 2f}$ feature matrix contains information about all self-attention mechanisms.

In order to enhance the representation of the data with the introduction of nonlinear modeling capabilities, the $H_{t\times 2f}$ is fed into a feedforward neural network. The feedforward neural network nonlinearly models the output multiple attention score matrices, where we use 1DCNN as a component of the feedforward neural network. The formula is as follows:

(10) $$I_{t\times 2f}=\mathrm{LayerNorm}(D_{t\times 2f}^{\left(m\right)}+H_{t\times 2f})$$

(11) $$Z_{\left(t,d\right)}=f_{1\mathrm{DCNN}}(I_{t\times 2f})=f\left(\sum_{i=0}^{k}I_{t\times k}\cdot W_{k\times 1}^{\left(d\right)}\right)$$

where t denotes the data at the tth moment in the window, d is the dimension after model mapping, $Z_{\left(t,d\right)}$ denotes the feature value after mapping to the multiple attention scores, $f_{1\mathrm{DCNN}}(\cdot )$ denotes that the model is mapped using a 1DCNN network, f(⋅) is the activation function, k denotes the size of the convolutional kernel, $H_{t\times k}$ represents the input polytope attention score selecting the k feature parameters at time t, and $W_{k\times 1}^{\left(d\right)}$ is the convolutional kernel weight parameter in the dth dimension of the output. By 1DCNN calculation, the output feature matrix can be expressed as:

(12) $$Z_{t\times d}=\left(\begin{array}{ccc} Z_{1,1} & \cdots & Z_{1,d}\\ \vdots & \ddots & \vdots \\ Z_{t,1} & \cdots & Z_{t,d} \end{array}\right)$$

Since the feedforward neural network contains two layers of 1DCNN, the $Z_{t\times d}$ is mapped back to its original dimension:

(13) $$S_{\left(t,2f\right)}=f_{1\mathrm{DCNN}}(Z_{t\times d})=\sum_{i=0}^{k}Z_{t\times k}\cdot W_{k\times 1}^{\left(f\right)}$$

where $S_{\left(t,2f\right)}$ is the output after feedforward neural network mapping; $Z_{t\times d}$ denotes the input feature matrix with k feature parameters selected at moment t, and $W_{k\times 1}^{\left(d\right)}$ is the output convolution kernel weight parameter in dimension d. Since the dimensions of $S_{\left(t,2f\right)}$ are different from the dimensions of the window data at the time of input, a fully connected layer is used to convert $S_{\left(t,2f\right)}$ to its original dimensions:

(14) $$S_{t\times f}=S_{t\times 2f}\times W_{2f\times f}+b_{t\times f}$$

where $W_{2f\times f}$ and $b_{t\times f}$ are the weights and bias of the fully connected layer, respectively, and $S_{t\times f}$ is the final output encoded with spatial features and $S_{t\times f}$ can be described as:

(15) $$S_{t\times f}=\left(\begin{array}{ccc} S_{1,1} & \cdots & S_{1,f}\\ \vdots & \ddots & \vdots \\ S_{t,1} & \cdots & S_{t,f} \end{array}\right)$$

#### 2.2.2. Temporal Feature Encoding

Temporal feature encoding is subsequently applied to the input data to capture sufficient temporal features, which uses parallels spatial feature coding, as it also employs a multi-head attention mechanism combined with a feedforward neural network. Temporal feature encoding is designed to map the temporal relationships within the input window data, focusing on capturing the relationships of the same parameter across different time points. This approach ensures the preservation and enhancement of temporal dependencies within the data, which are crucial for accurate anomaly detection in multivariate systems. Firstly, the input window data $D_{t\times 2f}^{\left(n\right)}$ is transposed to obtain the transposed data, $D_{t\times 2f}^{\left(n\right)}$ is inputted into Equations (6)–(11), and the feature matrix $H_{2f\times t}$ with all the information of the attention mechanism can be obtained from the output of the temporal feature encoding. The feedforward neural network in temporal feature encoding uses a linear layer as feedforward neural network, it is calculated as follows:

(16) $$T_{2f\times t}=f(H_{2f\times t}\times W_{t\times p}^{z}+b_{f\times p}^{z})\times W_{p\times t}^{z}+b_{2f\times t}^{z}$$

where $W_{t\times p}^{z}$ and $W_{p\times t}^{z}$ represent the weights of the two fully connected layers of the feedforward neural network species, p is the transformation dimension in the feedforward neural network, $b_{f\times p}^{Z}$ and $b_{2f\times t}^{z}$ denote the bias of the two fully connected layers of the feedforward neural network species, f(⋅) is the activation function, and $T_{2f\times t}$ denotes the output encoded with temporal features. In order to be able to convert $T_{2f\times t}$ to its original dimension, a fully connected layer is used to convert to the original dimension:

(17) $$T_{t\times f}={\left(S_{2f\times t}\right)}^{T}\times W_{2f\times f}+b_{t\times f}$$

where $W_{2f\times f}$ and $b_{t\times f}$ are the weights and biases of the fully connected layer, ${\left(\cdot \right)}^{T}$ is the transpose, and $T_{f\times t}$ denotes the output encoded with temporal features:

(18) $$T_{f\times t}=\left(\begin{array}{ccc} T_{1,1} & \ldots & T_{1,t}\\ \vdots & \ddots & \vdots \\ T_{f,1} & \ldots & T_{f,t} \end{array}\right)$$

#### 2.2.3. Spatio-Temporal Feature Fusion

UAV actuator data has complex spatio-temporal features. However, separate temporal feature extraction or spatial feature extraction cannot comprehensively capture the feature information in UAV actuator data. Through the fusion of temporal and spatial features, the temporal and spatial information in the data can be effectively synthesized to improve the model’s ability to characterize the correlation and temporal trend among the data, and the anomaly detection ability of the model can be effectively improved.

At this stage, the temporal features output from the previous stage with the spatial features are fused and passed to the encoding stage for final output. The output $S_{t\times f}$ obtained from spatial feature encoding is fused by multiplying it with the output $T_{t\times f}$ obtained from temporal feature encoding. Since the attention mechanism used in the above encoding process is able to learn the more important knowledge and ignore the unimportant knowledge, it is straightforward to fuse $S_{t\times f}$ with $T_{t\times f}$. In order not to destroy the shape of the original features, the Hadamard product method is used, and the obtained results are therefore parameterized so that the updating of the parameters therein can be achieved during the back-propagation process. Spatio-temporal features fusion is as follows:

(19) $$Parameter(St_{t\times f})=\left(\begin{array}{ccc} T_{1,1}\times S_{1,1} & \ldots & T_{1,f}\times S_{1,f}\\ \vdots & \ddots & \vdots \\ T_{t,1}\times S_{t,1} & \ldots & T_{t,f}\times S_{t,f} \end{array}\right)=\left(\begin{array}{ccc} T_{1,1} & \ldots & T_{1,t}\\ \vdots & \ddots & \vdots \\ T_{f,1} & \ldots & T_{f,t} \end{array}\right)\cdot \left(\begin{array}{ccc} S_{1,1} & \ldots & S_{1,f}\\ \vdots & \ddots & \vdots \\ S_{t,1} & \ldots & S_{t,f} \end{array}\right)$$

where $St_{t\times f}$ represents the spatio-temporal features obtained after fusion, ⋅ is the Hadamard product, $St_{t\times f}$ contains rich spatio-temporal features, and finally $St_{t\times f}$ is passed into the final decoding stage.

#### 2.2.4. Spatio-Temporal Feature Decoding

The $St_{t\times f}$ with rich spatio-temporal features is fed into the final target sequence, and the decoding phase contains two multi-head self-attention mechanisms and a feedforward neural network. Different from the traditional encoding stage of transformers, we introduce a $W_{1\times f}^{\left(n\right)}$ to promote the learning of the model, where $W_{1\times f}^{\left(n\right)}=\left[\begin{array}{llll} X_{t}^{1}, & X_{t}^{2}, & \ldots , & X_{t}^{f} \end{array}\right]$ is the last moment in the window data. The $St_{t\times f}$ and $W_{1\times f}^{\left(n\right)}$ are input into the encoding stage at the same time, and the detailed training process is as follows:

(20) $$Q_{i}=W_{1\times f}^{\left(n\right)}\times W_{f\times q}^{Q}+b_{1\times q}^{Q}$$

(21) $$K_{i}=W_{1\times f}^{\left(n\right)}\times W_{f\times q}^{K}+b_{1\times q}^{K}$$

(22) $$V_{i}=W_{1\times f}^{\left(n\right)}\times W_{f\times q}^{V}+b_{1\times q}^{V}$$

where q denotes the mapped feature dimension, and $Q_{i},K_{i},V_{i}$ are Query, Key, and Value from $W_{1\times f}^{\left(n\right)}$, respectively. $W_{f\times q}^{Q},W_{f\times q}^{K},W_{f\times q}^{V}$ represent the weight matrix of Query, Key, and Value, respectively, and $b_{t\times q}^{Q},b_{t\times q}^{K},b_{t\times q}^{V}$ denote the bias matrix of Query, Key, and Value, respectively. Using the obtained $Q_{i},K_{i},V_{i}$ with Equations (14)–(16), one can compute the long self-attention score matrix $H_{1\times f}=\left[\begin{array}{llll} H^{1}, & H^{2}, & \ldots , & H^{f} \end{array}\right]$ for $W_{1\times f}^{\left(n\right)}$. Later, the window sequence $W_{1\times f}^{\left(n\right)}$ is summed with the attention score matrix $H_{1\times f}$ to capture the temporal features and trends in the input sequence. The result of the summation is fed into the second multi-attention mechanism:

(23) $$I_{2}^{\left(n\right)}=\mathrm{MultiHeadAtt}(I_{1}^{\left(n\right)},St_{1\times f},St_{1\times f})$$

where $I_{2}^{\left(n\right)}$ denotes the output result after the second attention mechanism, $I_{1}^{\left(n\right)}$ is the result of adding the window sequence $W_{1\times f}^{\left(n\right)}$ with the attention score matrix, and MultiHeadAtt(⋅) denotes the multi-head attention mechanism. Finally, the final result is output using feedforward neural network:

(24) $$Y_{1\times f}^{\left(n\right)}=I_{1}^{\left(n\right)}+\mathrm{FeedForward}(I_{2}^{\left(n\right)})$$

where $Y_{1\times f}^{\left(n\right)}=\left[\begin{array}{llll} Y_{1\times f}^{\left(1\right)}, & Y_{1\times f}^{\left(2\right)}, & \ldots , & Y_{1\times f}^{\left(n\right)} \end{array}\right]$ denotes the final output after the decoding stage, and FeedForward(⋅) is the forward neural network.

#### 2.2.5. Self-Learning

To enhance the model’s capability to efficiently learn the temporal trends within the data window, we designed a self-learning module with two phases. In the first phase, the input data is reconstructed to generate a reconstruction window that closely resembles the input, and the reconstruction loss is calculated. In the second phase, the reconstruction loss obtained from the first phase is concatenated with the original training data, effectively augmenting the feature dimension. This allows the model to re-learn the input window data with additional context. During the transformer encoding stage, the internal multi-attention mechanism prioritizes subsequences with higher residual values, enabling the model to better focus on critical temporal trends. This approach enhances the model’s overall anomaly detection capability, the training data for the second phase is represented as:

(25) $$R_{t\times f}={\left|D_{t\times f}^{\left(n\right)}-Y_{1\times f}^{\left(n\right)}\right|}^{2}$$

where $R_{t\times f}$ is the reconstruction loss. Afterwards $D_{t\times f}^{\left(n\right)}$ is concatenated with $R_{t\times f}$ to form new data:

(26) $$O_{t\times 2f}^{\left(n\right)}=D_{t\times f}^{\left(n\right)}R_{t\times f}=\left(\begin{array}{cccccc} D_{11}^{\left(n\right)} & \ldots & D_{1f}^{\left(n\right)} & R_{11} & \ldots & R_{1f}\\ \vdots & \ddots & \vdots & \vdots & \ddots & \vdots \\ D_{t1}^{\left(n\right)} & \ldots & D_{tf}^{\left(n\right)} & R_{t1} & \ldots & R_{tf} \end{array}\right)$$

where $O_{t\times 2f}^{\left(n\right)}$ is the post-concatenation data. The $O_{t\times 2f}^{\left(n\right)}$ is again fed into the spatial feature coding, temporal feature coding and spatio-temporal feature decoding for training, and the final output is obtained as ${\hat{D}}_{t\times f}^{\left(n\right)}=\left[{\hat{D}}_{t,f}^{\left(n\right)},{\hat{D}}_{t,f}^{\left(n\right)},\ldots ,{\hat{D}}_{t,f}^{\left(n\right)}\right]$. The augmented representation is used as the input of the second-stage encoder, and the input projection layer of the encoder maps this augmented feature dimension to the hidden dimension required by the model.

### 2.3. Anomaly Detection

#### Anomaly Injection

In practice, collecting UAV anomaly data in real-world poses significant challenges. Intentionally inducing anomalies during flight carries a high risk of system failure or crashes, which may threaten public safety. Moreover, the high cost of UAV platforms means that improper handling of anomalies can result in substantial economic losses. Therefore, it is crucial to design detection parameters that can safely simulate typical UAV anomalies without compromising system integrity. The following section defines the bias anomalies used in this study:

(27) $$y_{\mathrm{bias}}(t)=y(t)+\alpha$$

where $y_{\mathrm{bias}}(t)$ represents the data after the occurrence of deviation anomaly. $y\left(t\right)$ is the original data, and α represents the deviation value. The stuck anomalies are defined as below:

(28) $$y_{\mathrm{stuck}}(t)=\beta$$

where $y_{\mathrm{stuck}}(t)$ denotes the anomaly data after the stuck occurred; β is the value at which the stuck occurred.

The proposed STF_Tran is trained for anomaly detection; the detection of anomalies relies on assessing whether the residuals, representing the deviation between the STF_Tran-reconstructed data and the actual input, surpass a specified threshold. The 3σ principle is used to select the threshold value.

For the input agnostic data $D_{t\times f}^{\left(n\right)}$, the output after model inference is ${\hat{D}}_{1\times f}^{\left(n\right)}$, so our anomaly score is defined as:

(29) $$S_{1\times f}^{\left(n\right)}={\left|{\hat{D}}_{1\times f}^{\left(n\right)}-D_{1\times f}^{\left(n\right)}\right|}^{2}$$

where $S_{1\times f}^{\left(n\right)}$ denotes the anomaly score. The $S_{1\times f}=S_{1\times f}^{\left(1\right)},S_{1\times f}^{\left(2\right)},\ldots ,S_{1\times f}^{\left(n\right)}$ can be obtained from a series of data. Then, the threshold for anomaly detection is as follows:

(30) $${\bar{S}}_{1\times f}=\frac{S_{1\times f}^{\left(1\right)}+S_{1\times f}^{\left(2\right)}+\ldots +S_{1\times f}^{\left(n\right)}}{n}$$

(31) $$\sigma_{1\times f}=\sqrt{\frac{1}{n}\sum_{n=0}^{n}{\left(S_{1\times f}^{\left(n\right)}-{\bar{S}}_{1\times f}\right)}^{2}}$$

(32) $$\Gamma_{1\times f}={\bar{S}}_{1\times f}+3\sigma_{1\times f}$$

where ${\bar{S}}_{1\times f}$ denotes the average anomaly score under normal data; $\sigma_{1\times f}$ is the standard deviation under normal data; $\Gamma_{1\times f}$ denotes the threshold value obtained. The selection of thresholds is performed on the validation set. For $\Gamma_{1\times f}=\{\Gamma_{1},\Gamma_{2},\ldots ,\Gamma_{f}\}$ there are f thresholds, corresponding to f parameters in the input data. For each anomaly score $S_{1\times f}^{\left(n\right)}$ is compared with $\Gamma_{1\times f}$, if $S_{1\times f}^{\left(n\right)}$ is higher than $\Gamma_{1\times f}$ then it is anomalous data, and if $S_{1\times f}^{\left(n\right)}$ is lower than $\Gamma_{1\times f}$ then it is normal data:

(33) $$\mathrm{Anomaly}=\left\{\begin{array}{ll} S_{1\times f}^{\left(n\right)}\geq \Gamma_{1\times f}, & \mathrm{True}\\ S_{1\times f}^{\left(n\right)}<\Gamma_{1\times f}, & \mathrm{False} \end{array}\right.$$

## 3. Dataset and Experimental Setup

### 3.1. Dataset Introduction

**Figure 3:**
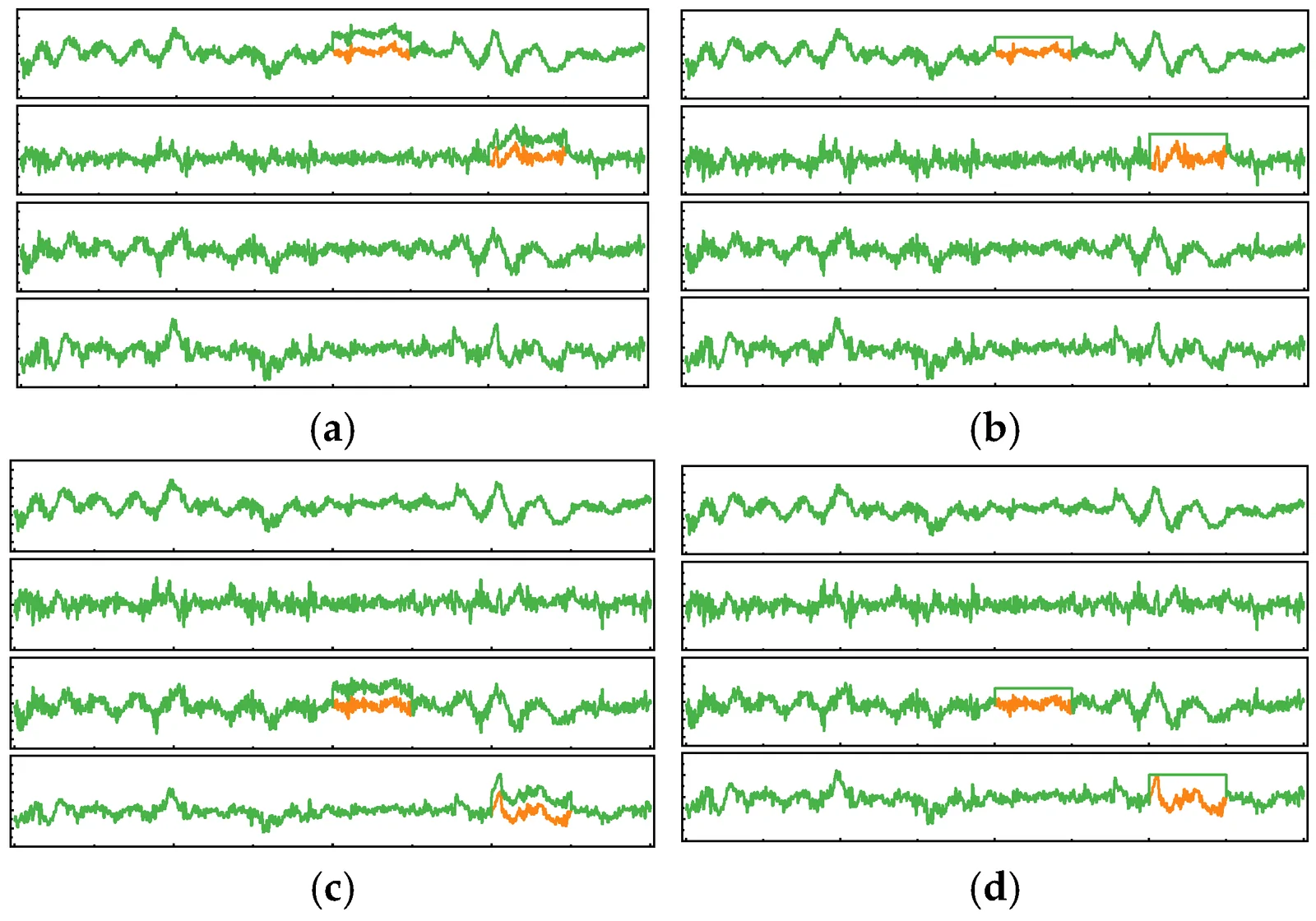
Anomaly injection. (**a**) Bias anomaly in Elevator and Aileron, (**b**) stuck anomaly in Elevator and Aileron, (**c**) bias anomaly in l_elevon and r_elevon, and (**d**) stuck anomaly in l_elevon and r_elevon. Orange lines means the anomalous fragment.

In this study, the dataset utilized is a public resource provided by the University of Minnesota Flight Laboratory, specifically selected from the 8th flight of the VIREO UAV. This dataset comprises parameters from six distinct UAV components, with the UAV data exhibiting clear spatial and temporal characteristics. The UAV’s rudder system, which governs flight direction, lift, and attitude through the coordinated operation of multiple actuators during flight, plays a pivotal role in UAV performance. Consequently, this study focuses on detecting anomalies in the actuator system. The detailed dataset parameters are summarized in Table 1. And Figure 3 illustrates the results of two experiments conducted with anomaly injection applied to the dataset. Therefore, the proposed framework is designed for multivariate actuator-level anomaly detection, where the coupled behavior among the Elevator, Aileron, left elevon, and right elevon is explicitly considered.

### 3.2. Evaluation Metrics

In this study, Mean Absolute Error (MAE) and Mean Squared Error (MSE) are utilized as key indicators for evaluating reconstruction performance, defined as follows:

(34) $$\mathrm{MAE}=\frac{1}{T-i}\sum_{t=i}^{T}\left(D_{t}-{\hat{D}}_{t}\right)$$

(35) $$\mathrm{MSE}=\frac{1}{T-i}\sum_{t=i}^{T}{\left(D_{t}-{\hat{D}}_{t}\right)}^{2}$$

where $D_{t}$ denotes the normal data, ${\hat{D}}_{t}$ is the output data, i is the point in time when the anomaly starts, and T represents the point in time when the anomaly ends. Lower values of MAE and MSE indicate better performance of the model. Anomaly detection focuses on the model’s ability to detect anomalies. In this study, F1, FPR, and ROC curves are used as indicators of anomaly detection as follows:

(36) $$\mathrm{Pre}=\frac{TP}{TP+FP}$$

(37) Re=TPTP+FN

(38) F1=2×Pre×RePre+Re

(39) $$\mathrm{FPR}=\frac{FP}{FP+TN}$$

TP (True Positive) and TN (True Negative) indicate correct identification of normal and anomalous samples, while FP (False Positive) and FN (False Negative) represent misclassifications. Precision and Recall are often in trade-off, and the F1 score is used to balance them, with higher values indicating better performance. The False Positive Rate (FPR) measures the proportion of normal samples incorrectly identified as anomalous; lower FPR means better detection. To evaluate performance across thresholds, the Receiver Operating Characteristic (ROC) curve plots TPR against FPR, and the Area Under the Curve (AUC) quantifies overall performance; a higher AUC reflects a more robust model.

In this study, the experimental analysis incorporates several statistical evaluation procedures. First, the actuator data are divided into training, validation, and testing subsets, and each actuator variable is normalized to reduce the influence of scale differences among variables. The validation subset is used to estimate the anomaly-score distribution under normal operating conditions, from which the mean and standard deviation are calculated for threshold determination. Second, reconstruction performance is quantitatively evaluated using MAE and MSE, which measure the average absolute and squared reconstruction deviations, respectively. Third, anomaly detection performance is evaluated using Precision, Recall, F1-score, FPR, ROC curves, and AUC, providing a statistical comparison of detection accuracy, false-alarm behavior, and threshold-independent classification capability. Furthermore, comparative experiments, ablation analysis, sliding window sensitivity analysis, and training data volume sensitivity analysis are conducted to evaluate the robustness and parameter sensitivity of the proposed method.

### 3.3. Comparison Methods

Most existing UAV anomaly detection methods focus on univariate detection, with relatively few studies addressing multivariate anomaly detection for UAVs. To comprehensively evaluate the performance of the methods proposed in this study, we conducted comparative experiments using several state-of-the-art multivariate anomaly detection approaches, including CAE-M [30], DAGMM [31], GDN [32], LSTM_AD [22,26], MSCRED [33], USAD [34], and AE-LSTM [18,23], alongside different fusion techniques such as STF_add and STF_nn. The parameter configurations for these comparison tests were based on those specified in the original papers. Detailed descriptions of the comparative methods are provided in Table 2.

### 3.4. Experimental Environment

In this study, the experimental environment is configured as follows: the system runs on Windows 11, with an Intel(R) Core (TM) i5-10400 CPU at 2.90 GHz, 64 GB of RAM, and an RTX 3090 graphics card. The model is implemented using the PyTorch 1.13 framework. During the training phase, the hyperparameters are set as follows: learning rate is 0.0001, number of epochs are 55, loss function is Mean Squared Error (MSE), optimizer is Adam, and dropout rate is 0.1.

## 4. Results Analysis

### 4.1. Comparison of Data Reconstruction Performance

**Figure 4:**
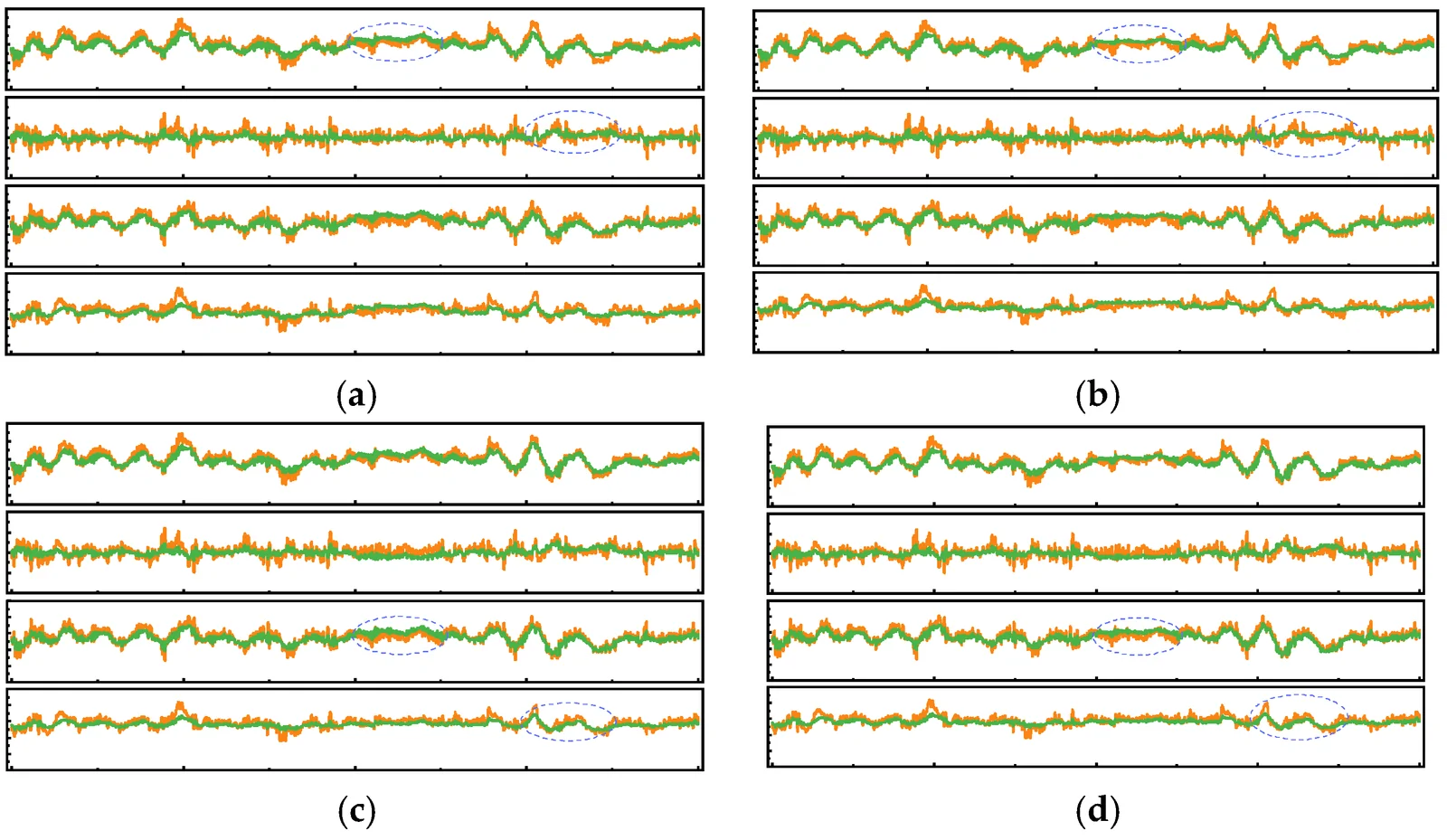
Data reconstruction performance of the STF_Tran. (**a**) Bias anomaly in Elevator and Aileron, (**b**) stuck anomaly in Elevator and Aileron, (**c**) bias anomaly in l_elevon and r_elevon, and (**d**) stuck anomaly in l_elevon and r_elevon. Orange lines means the reconstructed signals.

Figure 4 presents the data reconstruction visualization results of the STF_Tran, demonstrating its effectiveness in accurately reconstructing time periods with anomalies. To quantitatively evaluate the anomaly reconstruction, Table 3 summarizes the MAE and MSE values for data reconstruction on the different parameters using different methods, and the experimental results are calculated by averaging from five tests. The results indicate that DAGMM performs poorly, with MAE and MSE values reaching 0.1867 and 0.0443, respectively, on stuck anomalies. This underperformance is likely due to the Gaussian distribution assumptions of DAGMM, which limit its ability to capture the true data structure. LSTMAD and AE-LSTM perform better, with MSE values for deviation anomalies at 0.0032 and 0.0091, respectively. These methods show insensitivity to anomalous data, resulting in better reconstruction. USAD achieves the lowest MAE value of 0.0232 on stuck anomalies. For anomalies occurring on the left aileron (l_elevon), the performance difference between USAD and STF_Tran is minimal, while for the right aileron (r_elevon), the MAE and MSE differences are more pronounced, showcasing USAD’s strong reconstruction capabilities on l_elevon. MSCRED performs the worst, with MAE values for the two anomalies on r_elevon reaching 0.2563 and 0.1921, respectively. Conversely, STF_add exhibits excellent reconstruction performance on both Elevator and Aileron parameters, achieving an MSE value of 0.0015 on the Aileron. On the other hand, STF_NN excels in reconstructing the l_elevon parameter, achieving an impressive MSE value as low as 0.0027. These results highlight the strengths and weaknesses of different methods in reconstructing anomalies in UAV actuator data.

In summary, while USAD, MSCRED, DAGMM, and GDN achieve satisfactory reconstruction performance for certain parameters, they exhibit poor performance for others. This inconsistency is primarily due to their susceptibility to local optima during multivariate training, which leads to partial parameter fitting and an inability to comprehensively model the behavior of all parameters. In contrast, LSTMAD and AE-LSTM demonstrate more consistent and robust reconstruction across different parameters, reflecting better generalization. Notably, STF_Tran surpasses all other methods in overall reconstruction effectiveness. It consistently delivers superior results across diverse scenarios, further confirming the robustness and efficacy of the STF_Tran framework.

### 4.2. Anomaly Detection Performance Comparison

**Figure 5:**
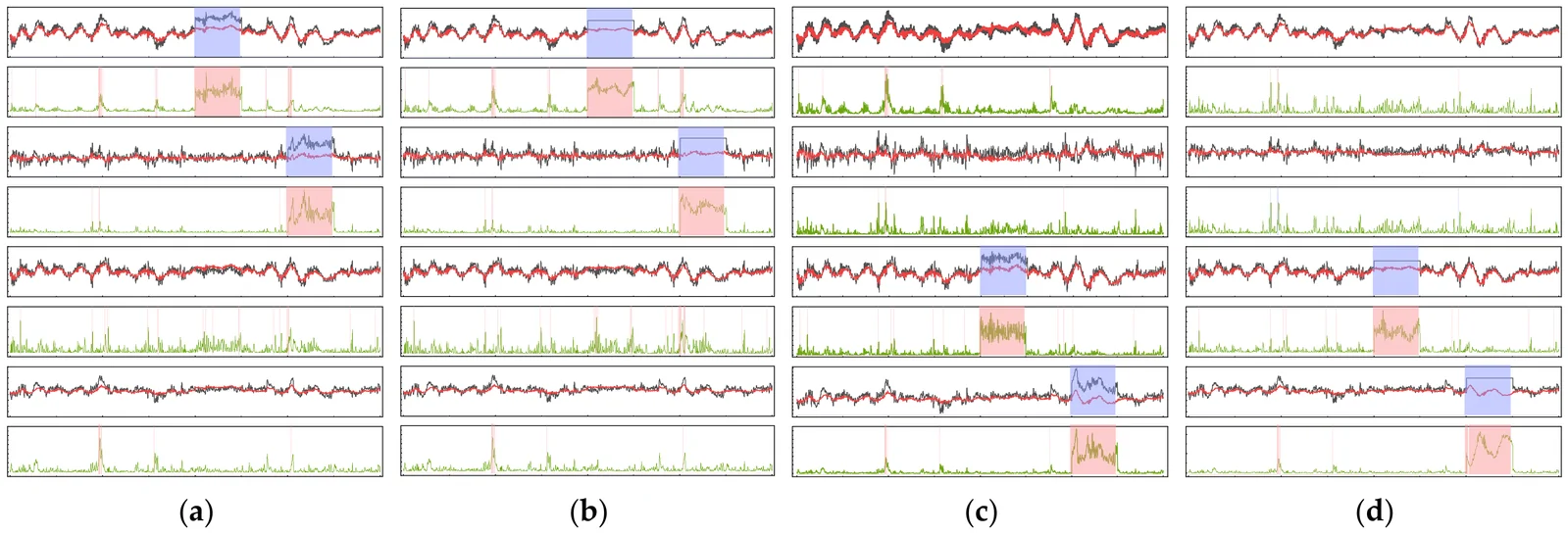
Visualization results of STF_Tran method for anomaly detection. (**a**) Bias anomalies in Elevator and Aileron, (**b**) stuck Anomaly in Elevator and Aileron, (**c**) bias anomalies in l_elevon and r_elevon, and (**d**) stuck Anomaly in l_elevon and r_elevon.

Figure 5 presents the visualization results of anomaly detection using the STF_Tran method. As illustrated, STF_Tran exhibits excellent data fitting capabilities and effectively detects both deviation and stuck anomalies. Although a small number of false positives appear within the normal data, STF_Tran achieves high precision in anomaly identification, demonstrating its robustness and reliability in handling diverse types of actuator anomalies.

Table 4 summarizes the anomaly detection results of STF_Tran compared with other methods. When anomalies were injected into the Elevator and Aileron, GDN and STF_Tran demonstrated the lowest FPR values, with FPRs of 0.0014 for both deviation and stuck anomalies, indicating strong anomaly detection capabilities. Among these, STF_Tran achieved the highest F1 scores, with 0.9923 and 0.9943 for bias and stuck anomalies. For anomalies injected into l_elevon and r_elevon, STF_Tran again showed superior performance, achieving F1 scores of 0.9952 and 0.9977 for deviation and stuck anomalies, respectively. The corresponding FPR values were 0.0145 and 0.0014. Although GDN achieved lower FPR values of 0.0012 for both types of anomalies, it fell short in F1 performance, highlighting STF_Tran’s overall balance between precision and recall. Regarding training efficiency, STF_Tran had the shortest training time per epoch, averaging 3.8265 s for training and 0.585 s for inferencing, whereas MSCRED had the longest training time, taking 272.014 s per epoch.

In general, GDN and MSCRED demonstrated strong FPR performance for both anomaly types but had low F1 values. This lower F1 performance can be attributed to a higher number of misdetections caused by anomalous variables influencing non-anomalous variables. Conversely, DAGMM and AE-LSTM achieved good F1 scores but exhibited high FPR values, suggesting these methods are better at fitting normal data but struggle to detect anomalies effectively. LSTMAD and CAE-M showed a mixed performance, excelling in F1 and FPR for l_elevon and r_elevon anomaly injections but suffering from numerous misdetections for Elevator and Aileron anomalies. Among the various fusion methods, models employing neural network fusion significantly underperformed compared to those using multiplicative fusion. The additive fusion approach demonstrated comparable performance to multiplicative fusion but with a notable increase in inference time. STF_NN required significantly longer training times than Tran_STF and STF_add, highlighting STF_Tran’s efficiency advantage in both detection accuracy and training time.

**Figure 6:**
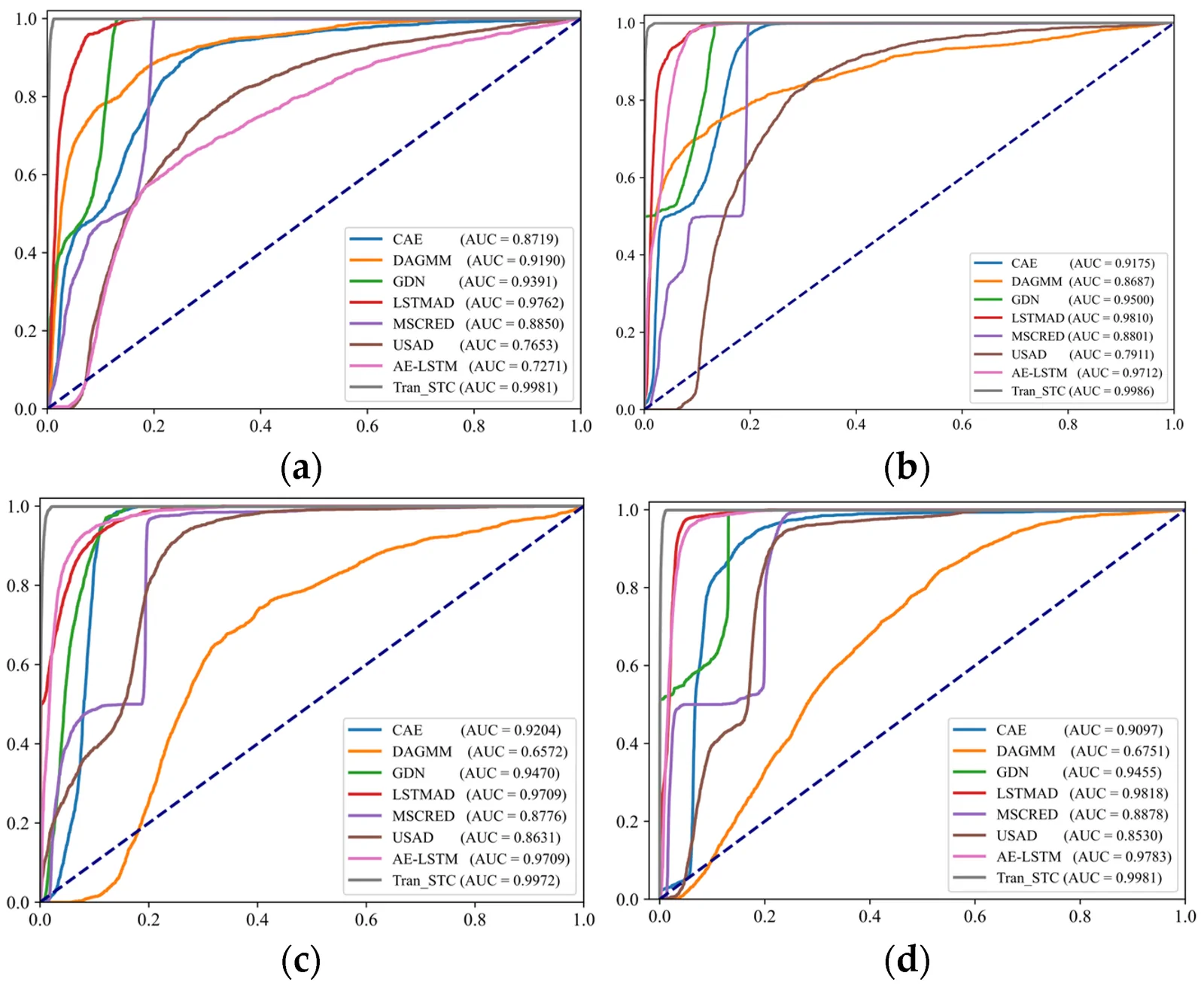
ROC curve for compared methods. (**a**) Stuck Anomaly in Elevator and Aileron, (**b**) bias anomalies in Elevator and Aileron, (**c**) stuck Anomaly in l_elevon and r_elevon, and (**d**) bias anomalies in l_elevon and r_elevon.

Figure 6 presents the ROC curves and AUC values for STF_Tran and other comparison methods. The results indicate that STF_Tran achieves AUC values exceeding 0.99 across four experiments, demonstrating superior anomaly detection performance. The LSTMAD method also performs well, with AUC values surpassing 0.97 in all experiments. Conversely, the CAE method exhibits lower AUC values across all tests, averaging around 0.9, but its performance remains consistent. The GDN method shows balanced results with an AUC value of approximately 0.95 across all experiments. However, some methods exhibit inconsistent performance. For instance, AE-LSTM achieves satisfactory results in most tests but falls short on the stuck anomalies for the Elevator and Aileron parameters, with an AUC value as low as 0.7271. Similarly, DAGMM displays strong results for Elevator and Aileron anomalies but performs poorly for l_elevon and r_elevon, indicating a limitation in generalizing across different parameters. The STF_nn method underperforms compared to STF_nn for the Elevator and Aileron parameters, but STF_add achieves higher AUC values for the r_elevon and R-elevator parameters than Tran_STF, although the differences are minor. Among the compared methods, STF_add demonstrates a distinct advantage in AUC values, emphasizing the efficacy of data fusion in enhancing multivariate anomaly detection for UAV actuators.

### 4.3. Ablation Analysis

To analyze the relative importance of the model’s components, we designed ablation experiments to evaluate the impact of each component on the performance metrics. The ablation configurations included the following scenarios: without temporal features (*w*/*o* T), without spatial features (*w*/*o* S), without the transformer module (*w*/*o* tr), and without the self-learning mechanism (*w*/*o* Self). By observing the effects of these components, we aimed to assess their individual contributions to the overall performance of the model.

#### 4.3.1. Ablation Analysis for Data Reconstruction

Table 5 presents the data reconstruction results under the ablation experiments. Overall, while all configurations yield relatively low MAE and MSE values, certain experiments exhibit significant differences in specific parameters. For the Elevator parameter, the absence of the self-learning module resulted in the lowest MAE and MSE values, at 0.0324 and 0.0016, respectively, for bias anomalies. Conversely, the Aileron and R-Elevon parameters achieved the lowest MAE and MSE values when all components of the model were included. Interestingly, the r_elevon parameter demonstrated the lowest MAE and MSE values for both stuck anomalies and bias anomalies when spatial features were excluded. These findings highlight the varying contributions of each component to the model’s reconstruction performance across different parameters.

#### 4.3.2. Ablation Analysis for Anomaly Detection

Table 6 presents the results of ablation experiments for deviation anomalies. From the table, it is evident that when anomalies are injected into the Elevator and Aileron parameters, models lacking spatial or temporal features exhibit relatively low F1 scores, reaching only about 0.95 in some cases. In contrast, the absence of the self-learning module has a minimal impact on the model’s performance, with differences in F1 values being as small as 0.001 and differences in FPR values only 0.002. For anomalies injected into the r_elevon and R-elevator parameters, the STF_Tran method demonstrates strong F1 performance. Notably, the FPR value for the model without temporal features is just 0.001, which is 0.002 lower than that of STF_Tran. Additionally, the model achieves its highest AUC value of 0.9994 when spatial features are excluded, highlighting the nuanced influence of individual components on the overall anomaly detection performance.

Table 7 presents the results of ablation experiments for stuck anomalies. From the table, it is evident that when anomalies are injected into the Elevator and Aileron parameters, the highest FPR value of 0.179 is observed in the absence of spatial features. However, other configurations demonstrate strong detection capabilities for stuck anomalies. The STF_Tran model achieves the highest AUC value of 0.9982, while the highest F1 score, 0.9912, is obtained in the absence of the self-learning module, which is only 0.001 higher than STF_Tran’s F1 score. For anomalies injected into the L-Elevon and R-Elevon parameters, STF_Tran delivers excellent performance, with F1 and AUC values reaching 0.9944 and 0.9972, respectively. Notably, when temporal features are excluded, the FPR achieves a low value of 0.008, further highlighting the nuanced contributions of temporal and spatial features to the model’s effectiveness in detecting stuck anomalies.

Combining the ablation experiments for bias and stuck anomalies reveals that different modules affect model performance to varying degrees. Excluding the transformer module significantly affects the FPR, with values reaching as high as 0.5005 and 0.3325 in certain experiments, indicating that the transformer module is crucial for anomaly sensitivity. The absence of temporal or spatial features primarily affects the F1 score, with some experiments showing F1 values around 0.95 when anomalies are injected into the Elevator and Aileron parameters. Overall, the STF_Tran method demonstrates consistently strong performance, with only minor variations in specific experiments, reaffirming its robustness and effectiveness.

## 5. Discussion

### 5.1. Window Size Analysis

To better analyze the impact of different sliding window sizes on model detection performance, we conducted experiments with sliding window sizes of 4, 12, 24, 32, 40, 48, and 56.

**Figure 7:**
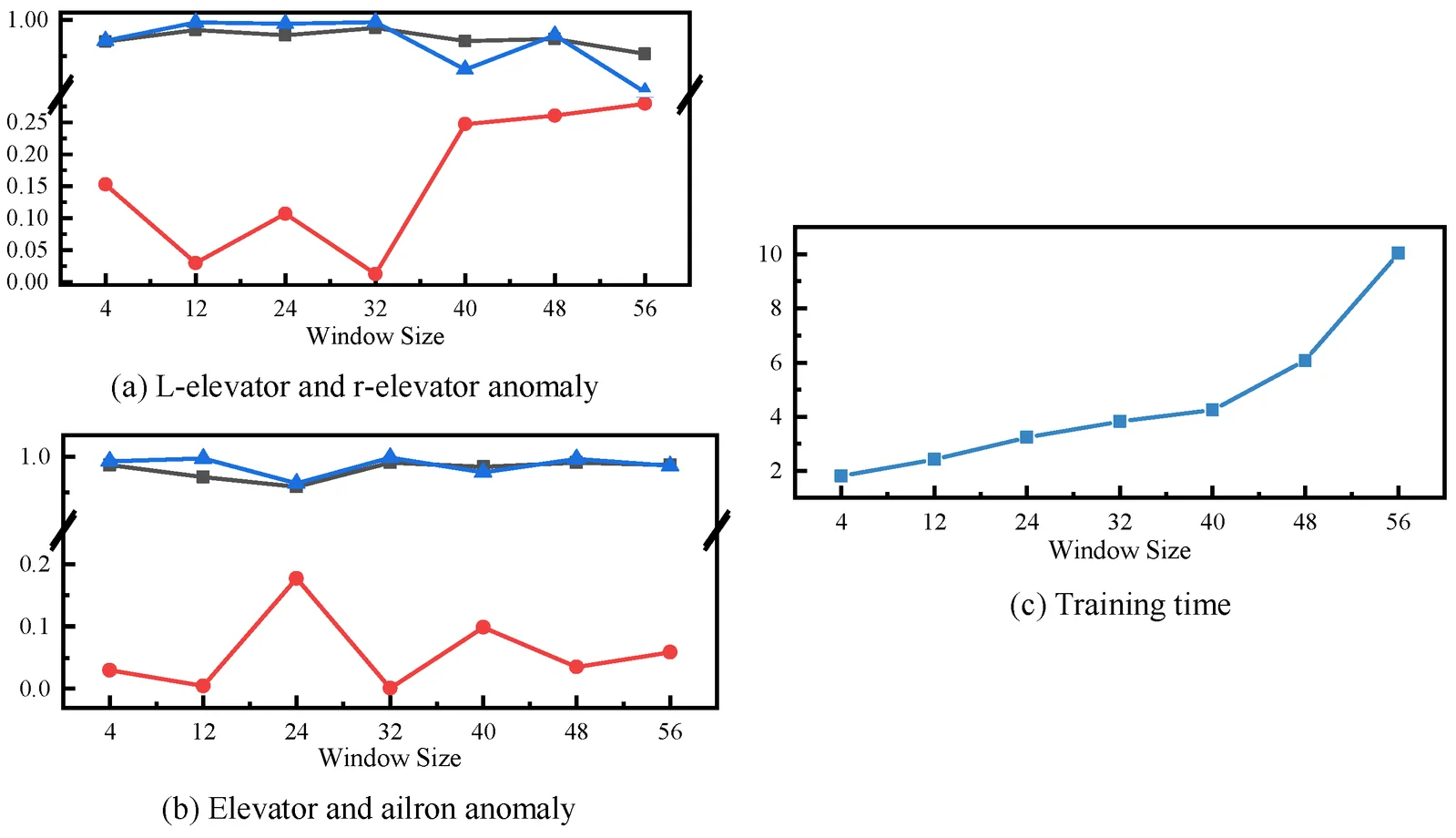
Model performance with different window sizes.

Figure 7 illustrates the model’s performance across various window sizes. When injecting anomalies into the r_elevon and R-elevator parameters, the F1 and AUC values initially increase as the window size grows. Notably, the FPR reaches 0.03 when the window size is 12. The model achieves its best performance at a window size of 32, with F1 and AUC values reaching 0.9944 and 0.9986, respectively, and the FPR decreasing to just 0.0125. However, as the window size exceeds 32, the model’s performance begins to decline, with the AUC dropping to 0.95 when the window size reaches 56. A similar trend is observed when anomalies are injected into the Elevator and Aileron parameters. At smaller window sizes, such as 4, 12, and 24, the model performs poorly, with F1 and AUC values of 0.9581 and 0.9621, respectively, at a window size of 24. The model achieves optimal performance at a window size of 32, with an FPR of only 0.001. However, as the window size increases beyond this point, the model’s performance deteriorates. In terms of training time, it increases as the window size grows. Up to a window size of 40, the training time rises gradually. Beyond this threshold, however, the training time increases more steeply, indicating a trade-off between window size and computational efficiency.

Table 8 presents the reconstruction performance of the model across different window sizes. The differences in MAE and MSE values are generally minor, but specific parameters achieve optimal reconstruction accuracy at certain window sizes. For the Elevator parameter, the lowest MAE and MSE values are observed at a window size of 4, with values of 0.0191 and 0.0006, respectively. The MSE value at a window size of 48 also reaches 0.0006. The Aileron parameter shows the best reconstruction at a window size of 32, with MAE and MSE values of 0.0292 and 0.0012. For the r_elevon parameter, the lowest MAE and MSE values occur at a window size of 24, recorded at 0.0095 and 0.0001, respectively. Similarly, the R-elevator parameter achieves its lowest reconstruction error at a window size of 32, with MAE and MSE values of 0.0324 and 0.0017. These results indicate that while the model’s reconstruction performance remains relatively stable across different window sizes, slight variations in accuracy are observed depending on the parameter and window size, where blue and black lines denote the detection AUC under different fault conditions; red lines represent reconstruction error (MAE/MSE), and the blue curve in subfigure (c) indicates model training time.

### 5.2. Sensitivity Analysis on the Size of Training Set

**Figure 8:**
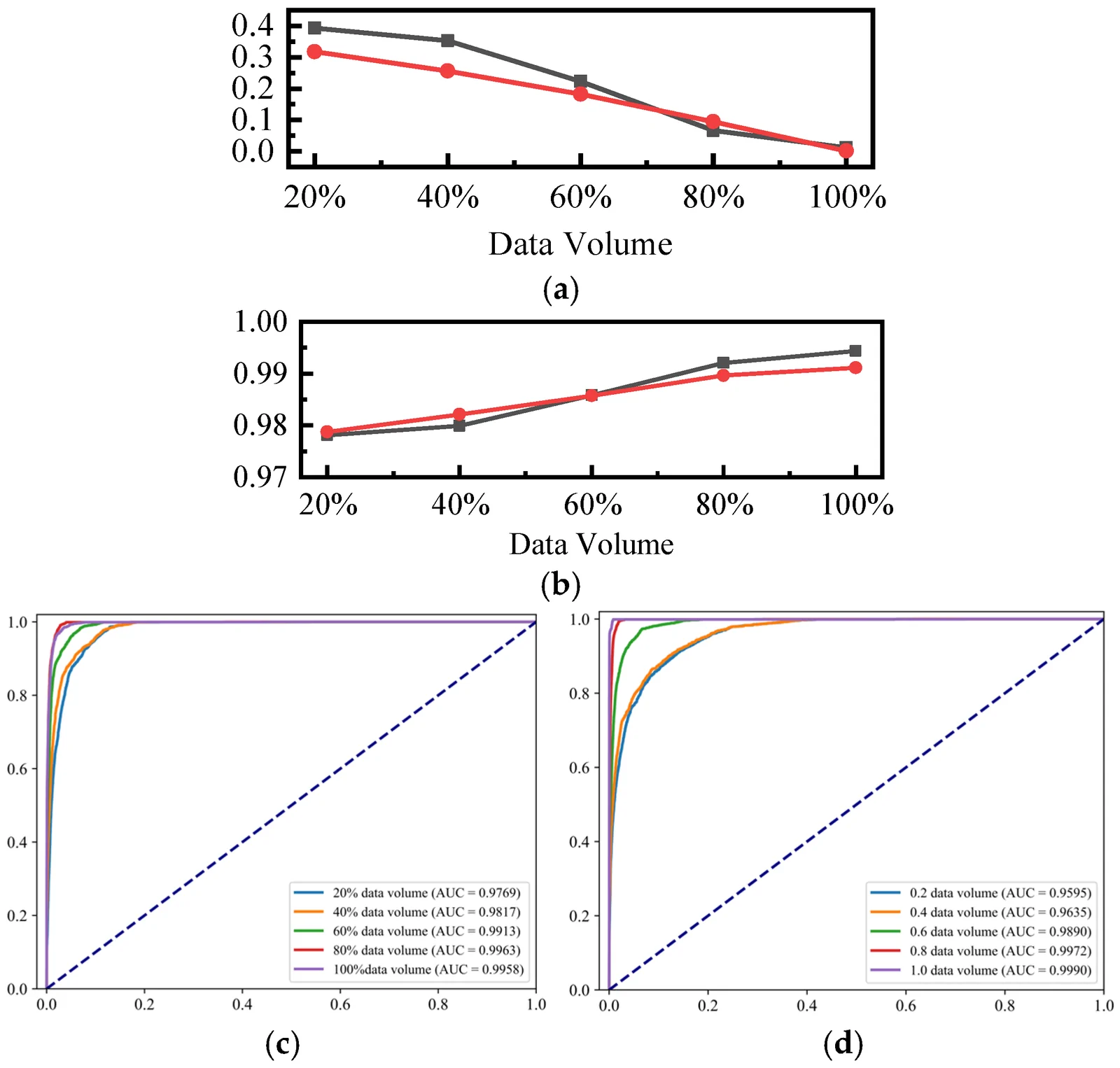
Indicators at different data size. (**a**) F1, (**b**) FPR, (**c**) Elevator, Aileron, and (**d**) l_elevon, r-elevon.

Figure 8 illustrates the performance metrics of STF_Tran across varying volumes of training data, highlighting the influence of data volume on model performance. In subplots (a) and (b), the black and red lines represent metrics for two different fault conditions, respectively. In subplots (c) and (d), the blue, orange, green, red, and purple ROC curves correspond to 20%, 40%, 60%, 80%, and 100% of the training data volume, while the dashed blue diagonal line denotes the random-guess baseline. As observed, the overall performance of the model improves progressively with an increase in training data. When the training data volume reaches 80%, the AUC index for Elevator and Aileron anomaly injections is slightly higher than that observed with 100% of the data, but the difference is negligible. Other performance indices demonstrate similar trends. This suggests that the volume of training data significantly affects model performance, particularly during initial increases. However, once the data volume exceeds a certain threshold, further additions have a diminishing impact on enhancing the model’s effectiveness.

Table 9 presents the data reconstruction performance of the model with varying data volumes. It is evident that the MAE and MSE values for the Elevator and l-elevon parameters are lower when the data volume is minimal compared to the case with 100% data.

**Figure 9:**
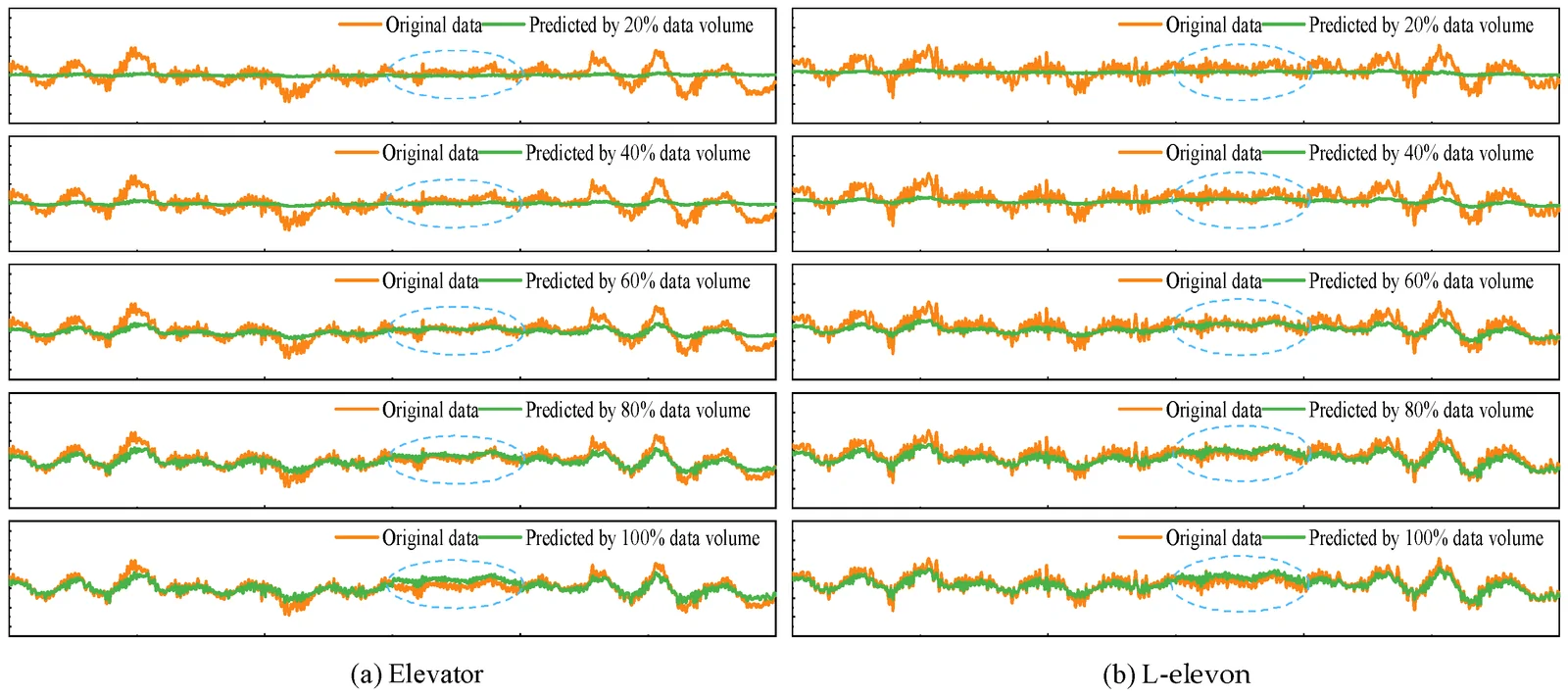
Fitted curves for different data volumes.

To explain this phenomenon, we visualized the results shown in Figure 9. When the data volume is small, the model does not effectively capture the temporal trends of the data, leading to output data that remains largely unchanged. Consequently, for injected anomalies within this time period, the output results are similar, resulting in relatively low MAE and MSE values. As the data volume increases, the model progressively learns the temporal trends. Additionally, with smaller data volumes, there is a noticeable disparity between the MAE and MSE values across different parameters, indicating that the model learns the features of each parameter unevenly. However, as the data volume grows, the gap between the MAE and MSE values of each parameter diminishes, demonstrating that the model achieves more balanced learning across all parameters. This highlights the importance of sufficient training data for consistent and accurate reconstruction performance.

### 5.3. Sensitivity Analysis on Window Size

Table 10 and Table 11 present the anomaly detection performance and data reconstruction results of the model under different window sizes. The findings indicate that the window size significantly influences both the detection capability and training time of the model. When the data within the window is minimal, the inference time is relatively short, with the model achieving a training time of 1.816 s. However, as the amount of data in the window increases, the inference time of the model fluctuates, showing gradual increases or decreases. This variability is attributed to the presence of noisy data within larger windows, which can adversely impact the model’s performance, as observed with a window size of 100. For a window size of 200, the window contains ample contextual information, which theoretically enhances the model’s performance. However, compared to smaller windows within the range of 4 to 54, the performance improvement is marginal, approximately 0.0015 in F1 for certain experiments. While specific parameters like Elevator and Aileron achieve MSE values of 0.0006 and 0.002, respectively, the model’s training time significantly increases to 45.646 s/epoch, while the estimated inference time is 1.014 ms/window. These results suggest that a window size in the range of 4 to 56 strikes a better balance between performance and computational efficiency, ensuring that the model meets both accuracy and time requirements effectively.

## 6. Conclusions

To achieve UAV actuator anomaly detection, this paper introduces an anomaly detection framework based on multivariate temporal and spatial feature fusion. First, two subnetworks are employed to extract temporal and spatial features from the data independently. Next, these features are fused to generate spatio-temporal features, which are subsequently decoded and output. Leveraging the transformer structure, the framework utilizes position encoding for single inference and GPU-based parallel operations, effectively capturing both temporal trends and spatial relationships in the data. This approach reduces training time while achieving multidimensional outputs. To validate the framework, stuck anomalies and bias anomalies were injected into real UAV actuator datasets. Comprehensive experiments demonstrate the following: (1) The proposed model significantly outperforms existing multivariate anomaly detection methods. (2) The method effectively reduces time complexity. (3) The impact of contextual information and training data volume on model performance is thoroughly analyzed.

Despite these promising results, this study has several limitations. The evaluation is based on data from the 8th flight of a single VIREO UAV, and thus the generalization of STF_Tran across different UAV platforms, flight conditions, and meteorological conditions remains to be validated. Future work will evaluate the framework across diverse UAV types and operating conditions, while further developing lightweight models and optimization methods for onboard real-time deployment [35].

## Figures and Tables

**Table 1:** Parameter description.

Name	Descriptions	Unit	Selection of Time	Train Length	Valid Length	Test Length
Elevator	Control of UAV pitch attitude and longitudinal stability	Deg	[70,000:100,000]	15,000	7000	8000
Aileron	Control of UAV aircraft roll attitude and lateral stability	Deg	[70,000:100,000]	15,000	7000	8000
l_elevon	Control the lift and roll of the UAV	Deg	[70,000:100,000]	15,000	7000	8000
r_elevon	Control the lift and roll of the UAV	Deg	[70,000:100,000]	15,000	7000	8000

**Table 2:** Methods of comparison.

Methods	Principle Overview
CAE-M	Combining an autoencoder with a CNN, anomaly detection is achieved by using reconstructed residuals compared to a threshold.
DAGMM	Combining an autoencoder with a Gaussian model, anomaly detection is achieved by using reconstructed residuals compared to a threshold.
GDN	A multivariate time-series anomaly detection method based on graph neural networks.
LSTM_AD	Using historical data to predict the next moment, the prediction residuals are compared with a threshold to achieve anomaly detection.
MSCRED	A Multiscale Convolutional Recursive Encoder–Decoder (MSCRED) for Anomaly Detection in Multivariate Time-Series Data
USAD	An autoencoder multivariate anomaly detection method based on inverse training.
AE-LSTM	Combining an autoencoder with LSTM, anomaly detection is achieved by using reconstructed residuals compared to a threshold.
STF_add	Similar to the STF_Tran method proposed in this paper, temporal features are integrated with spatial features during the data fusion phase.
STF_nn	Similar to the STF_Tran approach proposed in this paper, temporal features are concatenated with spatial features during the data fusion phase and then passed through a fully connected layer for output.

**Table 3:** Comparison of data reconstruction performance for different methods under injected anomalies.

Anomaly	Method	Elevator		Aileron		l_elevon		r_elevon	
		MAE	MSE	MAE	MSE	MAE	MSE	MAE	MSE
Bias	CAE-M	0.1618 ± 0.0522	0.03119 ± 0.0401	0.1132 ± 0.0314	0.0125 ± 0.0431	0.1422 ± 0.0540	0.0202 ± 0.0104	0.2135 ± 0.2014	0.0537 ± 0.1045
	DAGMM	0.1820 ± 0.0712	0.0516 ± 0.0921	0.1254 ± 0.0314	0.0312 ± 0.0917	0.0606 ± 0.0429	0.0107 ± 0.0575	0.1367 ± 0.0123	0.0254 ± 0.0712
	GDN	0.1327 ± 0.0991	0.0214 ± 0.4713	0.0609 ± 0.0875	0.0043 ± 0.0021	0.0533 ± 0.0720	0.0045 ± 0.9213	0.1915 ± 0.0402	0.0453 ± 0.1240
	LSTMAD	0.0556 ± 0.114	0.0102 ± 0.0213	0.0667 ± 0.0430	0.0075 ± 0.0021	0.0918 ± 0.0802	0.0083 ± 0.0043	0.1067 ± 0.0542	0.0178 ± 0.0124
	MSCRED	0.16811 ± 0.0909	0.0232 ± 0.0210	0.0923 ± 0.0312	0.0089 ± 0.0123	0.0727 ± 0.4012	0.0062 ± 0.0021	0.2563 ± 0.0323	0.0727 ± 0.0215
	AE-LSTM	0.0956 ± 0.0031	0.0101 ± 0.0342	0.0477 ± 0.0502	0.0062 ± 0.0421	0.0567 ± 0.0432	0.0053 ± 0.0084	0.1142 ± 0.0035	0.0212 ± 0.0073
	USAD	0.1896 ± 0.0643	0.0313 ± 0.0532	0.1522 ± 0.0432	0.0228 ± 0.0438	0.0453 ± 0.0910	0.0035 ± 0.0007	0.1545 ± 0.0901	0.0320 ± 0.0301
	STF_Tran	0.0388 ± 0.0306	0.0025 ± 0.0503	0.0298 ± 0.0301	0.0034 ± 0.0012	0.0483 ± 0.012	0.0034 ± 0.0012	0.0657 ± 0.0201	0.0078 ± 0.0012
Stuck	CAE-M	0.1560 ± 0.0092	0.0258 ± 0.0704	0.1167 ± 0.0092	0.0165 ± 0.0122	0.1012 ± 0.0304	0.0234 ± 0.0231	0.1475 ± 0.0065	0.0333 ± 0.0589
	DAGMM	0.1867 ± 0.1265	0.0443 ± 0.0407	0.1121 ± 0.0092	0.0198 ± 0.1209	0.04092 ± 0.0345	0.0043 ± 0.0041	0.0912 ± 0.0302	0.0089 ± 0.0021
	GDN	0.1321 ± 0.0043	0.0137 ± 0.0080	0.0789 ± 0.0328	0.0075 ± 0.0023	0.0599 ± 0.0223	0.0075 ± 0.0003	0.1335 ± 0.0013	0.0201 ± 0.0032
	LSTMAD	0.0529 ± 0.0012	0.0034 ± 0.0051	0.0839 ± 0.0021	0.0073 ± 0.0008	0.0798 ± 0.0012	0.0074 ± 0.0050	0.0711 ± 0.0034	0.0074 ± 0.0007
	MSCRED	0.1427 ± 0.0073	0.0234 ± 0.0071	0.1127 ± 0.0072	0.0132 ± 0.1245	0.0728 ± 0.0021	0.0039 ± 0.0091	0.1921 ± 0.0120	0.0237 ± 0.0012
	AE-LSTM	0.0894 ± 0.0213	0.0092 ± 0.0003	0.0478 ± 0.0121	0.0045 ± 0.0023	0.0591 ± 0.0023	0.0043 ± 0.0021	0.0611 ± 0.0021	0.0052 ± 0.0031
	USAD	0.1803 ± 0.0032	0.0418 ± 0.0021	0.1432 ± 0.0210	0.0232 ± 0.0134	0.0392 ± 0.0026	0.0034 ± 0.0014	0.0822 ± 0.0023	0.0092 ± 0.0017
	STF_Tran	0.0376 ± 0.0014	0.0020 ± 0.0123	0.0379 ± 0.0024	0.0021 ± 0.0037	0.0397 ± 0.0329	0.0027 ± 0.0120	0.0334 ± 0.0210	0.0022 ± 0.0212

**Table 4:** Anomaly detection results of comparative experiments.

Method	Anomaly	Elevator, Aileron		l_elevon, r_elevon		Train Time	Inference Time
		F1	FPR	F1	FPR		
CAE-M	Bias	0.9160 ± 0.0813	0.3455 ± 0.0482	0.9217 ± 0.0232	0.0410 ± 0.0.302	35.8583 ± 3.0124	0.797 ± 0.0420
	Stuck	0.9040 ± 0.0773	0.2059 ± 0.0016	0.9457 ± 0.1256	0.1356 ± 0.0291		
DAGMM	Bias	0.9431 ± 0.0023	0.9278 ± 0.0075	0.9561 ± 0.0092	0.7703 ± 0.0201	27.7088 ± 2.1017	0.616 ± 0.0021
	Stuck	0.9532 ± 0.0321	0.7435 ± 0.0017	0.9715 ± 0.0321	0.7402 ± 0.0324		
GDN	Bias	0.9185 ± 0.0025	0.0012 ± 0.0090	0.9210 ± 0.3021	0.0012 ± 0.0038	44.3695 ± 2.8914	0.986 ± 0.0038
	Stuck	0.9304 ± 0.0125	0.0020 ± 0.0035	0.9217 ± 0.2031	0.0020 ± 0.0009		
LSTMAD	Bias	0.9617 ± 0.0521	0.3235 ± 0.0021	0.9203 ± 0.0031	0.0019 ± 0.0083	10.1368 ± 0.4213	1.025 ± 0.0027
	Stuck	0.9642 ± 0.0324	0.1481 ± 0.0012	0.9704 ± 0.0025	0.0025 ± 0.0029		
MSCRED	Bias	0.8675 ± 0.0569	0.0025 ± 0.0031	0.8830 ± 0.0020	0.02551 ± 0.0203	272.014 ± 8.0328	5.045 ± 0.2019
	Stuck	0.8514 ± 0.0802	0.0025 ± 0.0032	0.8030 ± 0.0092	0.0451 ± 0.0059		
USAD	Bias	0.9122 ± 0.0321	0.6321 ± 0.0051	0.8897 ± 0.0026	0.4717 ± 0.0039	30.0767 ± 4.0091	0.628 ± 0.0022
	Stuck	0.9158 ± 0.0035	0.7281 ± 0.0201	0.8957 ± 0.0045	0.4243 ± 0.3002		
AE-LSTM	Bias	0.9527 ± 0.0312	0.9909 ± 0.0008	0.9804 ± 0.0094	0.6915 ± 0.0021	11.951 ± 2.1020	0.266 ± 0.0009
	Stuck	0.9682 ± 0.0201	0.7105 ± 0.1201	0.9803 ± 0.0321	0.7860 ± 0.1021		
STF_Tran	Bias	0.9923 ± 0.0302	0.0014 ± 0.0031	0.9952 ± 0.0037	0.0145 ± 0.0201	3.8265 ± 0.3012	0.585 ± 0.0102
	Stuck	0.9943 ± 0.0124	0.0014 ± 0.0033	0.9977 ± 0.0092	0.0014 ± 0.0008		

**Table 5:** Data reconstruction for ablation experiments.

	W/S	Elevator		Aileron		l_elevon		r-elevon	
		MAE	MSE	MAE	MSE	MAE	MSE	MAE	MSE
A/T	*w*/*o* S	0.1210	0.0149	0.0873	0.0083	0.0144	0.0003	0.0415	0.0026
	*w*/*o* T	0.0712	0.0052	0.0695	0.0055	0.0919	0.0085	0.0991	0.0101
	*w*/*o* tr	0.0385	0.0023	0.0454	0.0031	0.0409	0.0024	0.0661	0.0067
	*w*/*o* Self	0.0324	0.0016	0.0462	0.0029	0.0263	0.0011	0.0512	0.0037
	STF_Tran	0.0396	0.0019	0.0292	0.0012	0.0460	0.0025	0.0324	0.0017
	*w*/*o* S	0.1087	0.0127	0.0985	0.0114	0.0172	0.0005	0.0513	0.0034
	*w*/*o* T	0.0636	0.0045	0.0792	0.0078	0.0811	0.0070	0.1149	0.0148
	*w*/*o* tr	0.0385	0.0023	0.0454	0.0031	0.0409	0.0024	0.0661	0.0066
	*w*/*o* Self	0.0375	0.0021	0.0528	0.0038	0.0295	0.0014	0.0759	0.008
	STF_Tran	0.0354	0.0018	0.0349	0.0018	0.0387	0.0021	0.0324	0.0017

**Table 6:** Ablation experiments for bias anomalies.

Method	Elevator, Aileron			l_elevon, r_elevon		
	F1	FPR	AUC	F1	FPR	AUC
*w*/*o* S	0.9575	0.175	0.9322	0.9877	0.012	0.9994
*w*/*o* T	0.9515	0.001	0.9994	0.9419	0.001	0.9961
*w*/*o* tr	0.9790	0.3325	0.9760	0.9754	0.458	0.9532
*w*/*o* self	0.9866	0.0865	0.9848	0.9666	0.224	0.9605
STF_Tran	0.9927	0.001	0.9986	0.9949	0.003	0.9981

**Table 7:** Ablation experiments for stuck anomalies.

Method	Elevator, Aileron			l_elevon, r_elevon		
	F1	FPR	AUC	F1	FPR	AUC
*w*/*o* S	0.9597	0.179	0.9291	0.9842	0.0475	0.9962
*w*/*o* T	0.9517	0.001	0.9953	0.9488	0.008	0.9762
*w*/*o* tr	0.9899	0.001	0.9853	0.9741	0.5005	0.9830
*w*/*o* self	0.9888	0.001	0.9921	0.9704	0.009	0.9794
STF_Tran	0.9911	0.001	0.9982	0.9944	0.0125	0.9972

**Table 8:** Data reconstruction in different windows.

W/S	Elevator		Aileron		l_elevon		r_elevon	
	MAE	MSE	MAE	MSE	MAE	MSE	MAE	MSE
4	0.0191	0.0006	0.0500	0.0031	0.0535	0.0032	0.0535	0.0041
12	0.0323	0.0013	0.0439	0.0024	0.0382	0.0016	0.0506	0.0033
24	0.0707	0.0053	0.0750	0.0067	0.0095	0.0001	0.0529	0.0038
32	0.0396	0.0019	0.0292	0.0012	0.0460	0.0025	0.0324	0.0017
40	0.0293	0.0013	0.0714	0.0059	0.0410	0.0019	0.0696	0.0067
48	0.0214	0.0006	0.0398	0.0021	0.0571	0.0037	0.0534	0.0038
56	0.0263	0.0011	0.0521	0.0033	0.0742	0.0057	0.0662	0.0061

**Table 9:** Data reconstruction with different data volumes.

D/V	Elevator		Aileron		l_elevon		r_elevon	
	MAE	MSE	MAE	MSE	MAE	MSE	MAE	MSE
20%	0.0356	0.0020	0.0460	0.0033	0.0458	0.0030	0.0664	0.0065
40%	0.0323	0.0017	0.0461	0.0032	0.0409	0.0024	0.0647	0.0061
60%	0.0204	0.0007	0.0421	0.0027	0.0256	0.0009	0.0497	0.0037
80%	0.0266	0.0009	0.0373	0.0020	0.0266	0.0010	0.0398	0.0025
100%	0.0396	0.0019	0.0292	0.0012	0.0460	0.0025	0.0324	0.0017

**Table 10:** Anomaly detection results in different windows.

W/S	Elevator, Aileron			l_elevon, r-elevon			Train Time	Inference Time
	F1	FPR	AUC	F1	FPR	AUC		
4	0.9879	0.03	0.9931	0.985	0.153	0.9855	1.816	0.044
56	0.988	0.059	0.9866	0.9763	0.279	0.9657	10.029	0.235
100	0.8937	0.3865	0.8045	0.9609	0.001	0.9992	15.09	0.337
200	0.9894	0.104	0.9912	0.9923	0.0065	0.9962	45.646	1.014

**Table 11:** Anomaly detection results under different windows.

W/S	Elevator		Aileron		l_elevon		r-elevon	
	MAE	MSE	MAE	MSE	MAE	MSE	MAE	MSE
4	0.0191	0.0006	0.0500	0.0031	0.0535	0.0032	0.0535	0.0041
56	0.0263	0.0011	0.0521	0.0033	0.0742	0.0057	0.0662	0.0061
100	0.1853	0.0345	0.1472	0.0217	0.0456	0.0021	0.0083	0.0001
200	0.0207	0.0006	0.0412	0.002	0.0854	0.0082	0.0321	0.0015

## Data Availability

The data presented in this study are available on request from the corresponding author.
